# Ischemic strokes in COVID-19: risk factors, obesity paradox, and distinction between trigger and causal association

**DOI:** 10.3389/fneur.2023.1222009

**Published:** 2023-08-01

**Authors:** Francesco Janes, Emanuela Sozio, Gian Luigi Gigli, Andrea Ripoli, Francesco Sbrana, Fedra Kuris, Lorenzo Nesi, Tosca Semenzin, Giacomo Bertolino, Cristian Deana, Daniele Bagatto, Chiara Ciardi, Martina Fabris, Giovanni Merlino, Francesco Bax, Annacarmen Nilo, Sara Pez, Mariarosaria Valente, Carlo Tascini

**Affiliations:** ^1^Clinic of Neurology, Azienda Sanitaria Universitaria Friuli Centrale (ASUFC), Udine, Italy; ^2^Department of Medical Area (DAME), University of Udine (UNIUD), Udine, Italy; ^3^Clinic of Infectious Diseases, Azienda Sanitaria Universitaria Friuli Centrale (ASUFC), Udine, Italy; ^4^Cardiology OU, Cardiothoracic Department, G. Monasterio Foundation, Pisa, Italy; ^5^Department of Anesthesia and Intensive Care, Azienda Sanitaria Universitaria Friuli Centrale (ASUFC), Udine, Italy; ^6^Institute of Neuroradiology, Azienda Sanitaria Universitaria Friuli Centrale (ASUFC), Udine, Italy; ^7^Institute of Clinical Pathology, Azienda Sanitaria Universitaria Friuli Centrale (ASUFC), Udine, Italy

**Keywords:** COVID-19, acute stroke, acquired coagulopathy, triggers of acute stroke, obesity paradox

## Abstract

**Background and purpose:**

Stroke has been described as a COVID-19 complication. However, its occurrence rate, risk factors, and causal relationships are still not well established.

**Methods:**

We describe the characteristics of confirmed COVID-19-related strokes among all cases of COVID-19 hospitalized in our health network, from November 1, 2020 to April 30, 2021. Risk factor analysis has been conducted for ischemic stroke (IS), which represents 92% of all confirmed cases of Covid-19-related strokes, and a “causal attribution to infection” classification is provided.

**Results:**

In all, 62/4105 hospitalized COVID-19 patients had an acute stroke (1.51%). Severe COVID-19 (OR 2.27—CI 1.06–4.77; *p* = 0.032), atrial fibrillation (OR 3.65—CI 1.63–7.98; *p* = 0.001), and ischemic heart disease (OR 4.590—CI 1.714–12.137; *p* = 0.002) proved to be independent risk factors for IS, while obesity was a protective factor (OR 0.90—CI 0.82–0.97; *p* = 0.012). COVID-19 had a causal role in 32.1% of IS cases, was a relevant cofactor in 28.6% of cases of IS, and was a possible trigger in 39.3% of events.

**Conclusion:**

Our stroke occurrence rate is consistent with other population-based reports (range 0.34–2.7%). Prespecified peculiar clinical and radiological features allow the distinction between “IS caused by COVID-19” and “IS triggered by COVID-19.” Clinical history of vascular diseases and risk factors is crucial in determining the risk of IS in patients with COVID-19. However, the protective effect of a BMI > 30 kg/m^2^ seems to suggest an obesity paradox.

## Highlights

- Stroke is a complication of COVID-19 in 1.51% of hospitalized patients.
- In COVID-19 patients, 32.1% of strokes are likely to be caused by infection-induced coagulopathy, while 67.9% are probably or possibly triggered by viral infection.
- COVID severity, atrial fibrillation, and ischemic heart disease are independent risk factors for acute stroke in COVID-19 patients.
- Higher BMI and obesity paradoxically proved to protect COVID-19 patients from ischemic stroke occurrence.
- The independent association of the most severe COVID-19 forms with stroke occurrence encourages the need for effective and long-lasting vaccination against COVID-19.

## Introduction

After 3 years of the ongoing pandemic, strokes have been extensively described as a possible complication of COVID-19. Acute infection with SARS-CoV-2 seems to confer a risk of stroke up to 7-fold higher than previously described with influenza A and B (1, 2). Strokes related to COVID-19 proved to be characterized by some distinctive clinical, laboratory, and instrumental features if compared to overall acute strokes. Acute ischemic stroke (AIS) is largely the most common and studied stroke subtype in COVID-19 patients. The most relevant features that have been highlighted in the literature are a higher proportion of large artery disease (LAD) (3) and multifocality (4, 5), a younger age at onset (6), a higher rate of concurrent systemic thrombotic events (7), resistance to prophylactic and even therapeutic antithrombotic therapy (8–10), a higher NIHSS at onset, and a worse functional outcome (11). A lower number of reports described intracerebral hemorrhage (ICH) related to COVID-19 infection, pointing out the more inhomogeneous pattern of presentation at neuroimaging, the higher tendency to early hematoma expansion (12), the high mortality rate, COVID-19 severity as a risk factor, and the role of therapeutic anticoagulation as an independent risk factor for ICH (13, 14).

Thus far, despite the large number of research papers published in the last 2 years, there are still some uncertainties about the occurrence/incidence rate of strokes in COVID-19 and the clinical profile of the patient with COVID-19 infection that is “at risk” for stroke occurrence. In cohorts of COVID-19 patients, stroke occurrence has been reported globally to range from 0.1 to 25.7% (15–25). This high variability is explained above all by the high heterogeneity of different hospital settings, referrals’ codes, and the cohorts’ characteristics. In the few population-based studies available, stroke occurrence/incidence ranges from 0.34 to 2.7% of hospitalized patients with COVID-19 (19, 26–28). COVID-19 severity (26) and conventional vascular risk factors (28) have been associated with AIS incidence. In only one study, a prespecified distinction between a triggering role of COVID-19 and a causal one was proposed, with authors observing that “COVID-19 was the likely principal cause of stroke in 24% of patients” (4). After a complete stroke diagnostic workup, Requena et al. (23) discussed that <40% of their AIS cases were probably related to COVID-19.

Some consensus is emerging that the impact of COVID-19 is destined to influence stroke research and epidemiology even beyond the first impetuous waves of the ongoing pandemic (29, 30). So, additional data on stroke occurrence in COVID-19 and on the patients at risk of cerebrovascular complications are extremely important. More data are also relevant to outline the relationship between systemic inflammation/infection and cerebrovascular disease, both as a trigger of and a direct cause of the event itself.

In this study, we aimed at (i) providing additional data on stroke occurrence during a COVID-19 infection; (ii) depicting risk factors for stroke in patients with a COVID-19 infection; and (iii) defining boundaries among causal, cofactor, or incidental roles in the relationship between COVID-19 and acute stroke.

## Methods

### Patients’ selection

We acquired data from all the patients hospitalized in the hospital network of our institution, covering a population of 526.474 inhabitants (31), from November 1, 2020 to April 30, 2021. The hospital system in our metropolitan area consists of one hub hospital and five local spoke hospitals. The dedicated Neurocovid ward and Infectious Disease Clinic are hosted in the hub facility only, whereas intensive care units (ICUs) and medical wards were also available in all the spoke centers.

**Figure 1:**
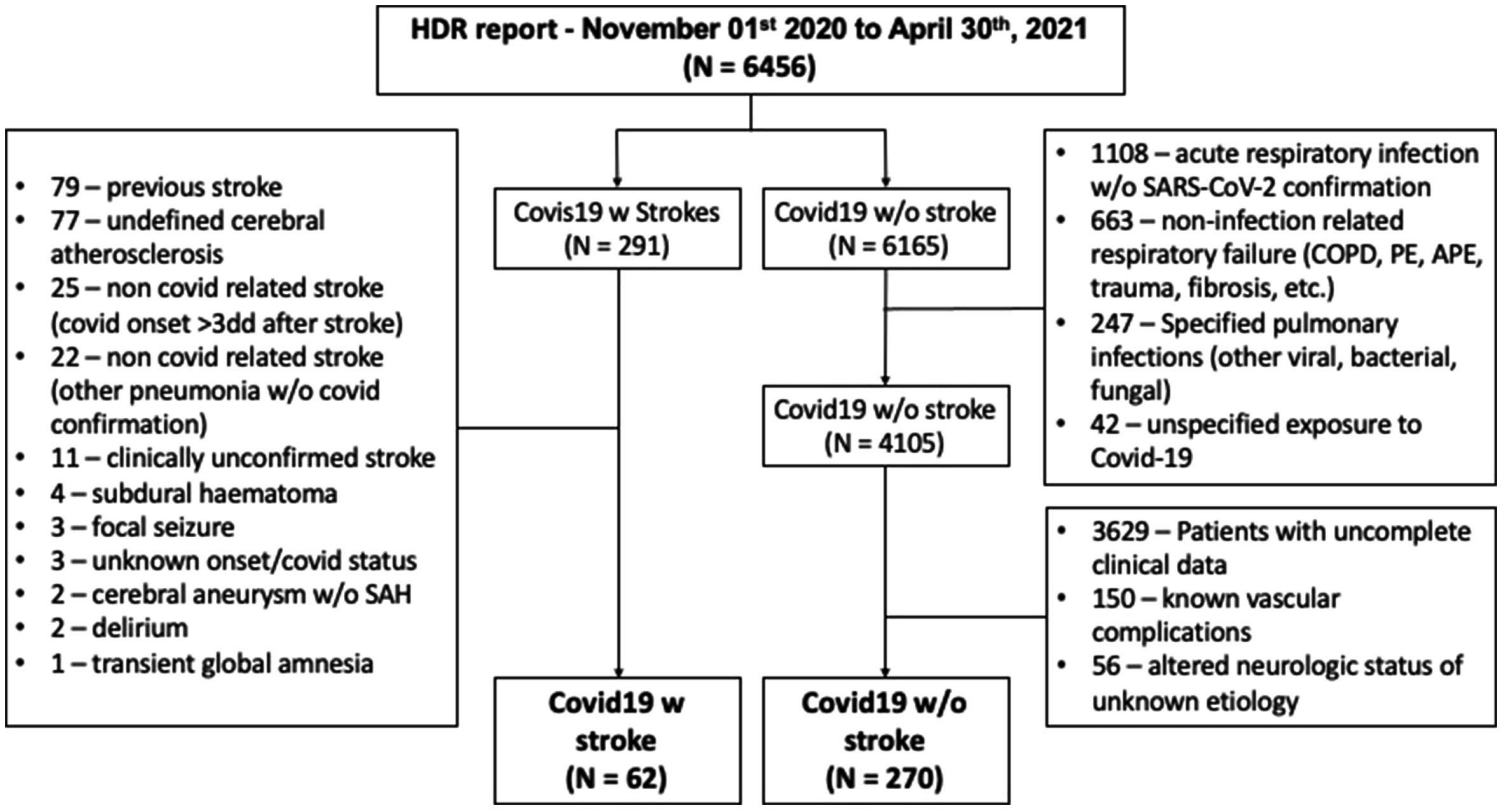
Patients’ selection process. HDR, hospital discharge records; SAH, subarachnoid hemorrhage; COPD, chronic obstructive pulmonary disease; PE, pulmonary embolism; and APE, acute pulmonary edema.

We selected patients who had in their Hospital Discharge Records (HDRs) a diagnosis of respiratory infection associated with COVID-19 (ICD9 codes: 079.82, 079.99, 043.xx, 480.xx, 518.xx, and 519.xx). HDRs were reviewed carefully by one of the authors (FJ) and duplicated records and wrong codifications in both COVID-19 and stroke diagnoses were excluded. From the initial 6,456 records with a COVID-19-related diagnosis, 291 had a stroke-related diagnosis (ICD9 codes: from 430.xx to 438.xx) and 4,105 records had a confirmed COVID-19 infection. After revision of the HDR, 62 patients with a diagnosis of acute stroke associated with COVID-19 infection were left for analysis. In the group without stroke, after the exclusion of patients with incomplete/unavailable clinical data and those with known vascular complications, 270 patients were finally selected as the control group. Figure 1 shows the details of this selection process.

### Diagnosis and risk factors definitions; classification systems and scores

Stroke was defined as an acute onset of symptoms and/or signs of focal disturbance of cerebral function lasting >24 h or leading to death, presumed, after exclusion of other causes, to be due to vascular disease (32). Patients who received reperfusion therapies were diagnosed with stroke even if symptoms were completely resolved within 24 h. Incident stroke was defined as a first-ever-in-a-lifetime (FES) stroke, occurring within the study period and with no clinical history of previous stroke.

The diagnosis of COVID-19 infection was confirmed in all patients through reverse transcriptase viral PCR testing. Patients whose test became positive >72 h after admission or stroke onset were excluded from this series.

The vascular diseases, other than stroke, considered in the patients’ selection process (and reported as “concurrent vascular events” in the stroke group) included pulmonary embolism (PE), deep/superficial venous thrombosis (D/SVT), acute myocardial infarct (AMI), and acute intestinal infarction (AII).

The Trial of ORG 10172 in Acute Stroke Treatment (TOAST) criteria were used to classify ischemic stroke etiology into the following seven groups: large artery disease (LAD), cardioembolic (CE), small vessel disease (SVD), undetermined strokes because of two or more causes identified (UND-a), undetermined strokes because of negative evaluation (UND-b), undetermined strokes because of incomplete workup (UND-c), and other determined etiology (OTH) (33). Stroke due to a large vessel occlusion (LVO) was defined if there was evidence of occlusion/severe stenosis of an extracranial epiaortic artery, intracranial internal carotid artery, and middle cerebral artery (both M1 and M2 tracts). Evidence of aortic atheroma >1.5 mm was considered qualifying for a large vessel stenosis/occlusion and LAD in the TOAST category. Carotid stenosis was measured by either cerebral tomographic angiogram (CTA), neck, or transcranial ultrasound according to NASCET or ECST criteria and classified as mild (0–30% stenosis), moderate (31–50% stenosis), severe (51–70%), and critical (71%-occlusion).

High blood pressure was defined as systolic pressure of 140 mmHg and/or diastolic pressure of 90 mmHg, and/or the use of antihypertensive medication. Atrial fibrillation was diagnosed if a patient had atrial fibrillation in EKG recording before stroke and/or during hospitalization. Diabetes mellitus was defined as a history of diabetes that was confirmed in medical records, and/or use of insulin/oral hypoglycemic agents, and/or random non-fasting blood glucose concentration of 11.1 mmol/L. Hypercholesterolemia was defined as a fasting total cholesterol serum level of 5.18 mmol/L (200 mg/dL), and/or fasting low-density lipoprotein cholesterol serum level of 4.14 mmol/L (160 mg/dL), and/or use of lipid-lowering medications. Coronary heart disease was defined as a history of acute myocardial infarction, angina pectoris, coronary artery bypass graft, or percutaneous coronary intervention. Patients were defined as smokers if they were current smokers, or if they had stopped smoking less than 3 months before the index stroke/TIA. Alcohol consumption was defined as “overuse” if a patient reported taking regularly more than two alcoholic units per day. Obesity was herein considered according to the most accredited global definition as a BMI > 30 kg/m^2^ (34).

The van-Swieten score was used to grade concurrent chronic vascular encephalopathy or leukoaraiosis on brain CT scans (35). The NIHSS and the modified Rankin Scale (mRS) were used as markers of stroke severity and outcome. COVID-19 severity was classified according to the WHO severity index (0 = asymptomatic; 1 = mild with no pneumonia; 2 = moderate pneumonia; and 3 = ARDS and critically ill) (36).

### Association of ischemic stroke with COVID-19 infection

We defined three levels of likelihood of association, regardless of the TOAST assignment, and according to the patient’s vascular conventional risk factor (RF) profile and the stroke-specific features (SSF) indicating an active hyper-coagulopathy (i.e.: multifocality of brain lesions, associated signs of acute/subacute atherosclerosis at vascular imaging, concurrent extracerebral thrombotic events, and occurrence despite adequate antithrombotic therapy):

1. Causal association: COVID-19 infection is likely the main determinant of ischemic stroke (the patient has <2 known RFs and > 2 SSF).
2. Probable trigger/concurrent factor: the patient’s clinical history and risk factor profile suggest a preexisting moderate to high risk of stroke, but atypical stroke features indicate a probable role of acute infection (the patient has >2 known RFs and at the same time > 2 SSF).
3. Possible trigger/concurrent factor: the patients’ clinical history and risk factor profile were already coherent with stroke occurrence (the patient has >2 known RFs and < 2 SSF), and COVID-19 infection had a low probability of being a key element in ischemic stroke pathogenesis.

### Statistical analysis

Continuous variables are described as mean ±± standard deviation (sd), and an unpaired *t*-test was used to compare groups. Categorical variables are shown as percentages out of the total and the Chi2 test was used to compare groups. Univariate analysis and multiple logistic regression were used to identify independent risk factors associated with stroke occurrence. The relationship between the investigated variables was studied with a network analysis approach. Continuous variables were discretized to allow network analysis (e.g.: BMI became obesity according to the WHO definition). The association between each couple of variables was assessed by the non-parametric and non-linear method of mutual information; the structure of the network was then computed with the ARACNE algorithm. Given the small sample size, the uncertainty of the associations was assessed with bootstrap (37). Data were analyzed using spss 29.0 (IBM corporation, New York, United States, 2022).

### Ethics

The study was approved by the regional ethics committee as part of the Sarscov2 infection Stroke Study initiative—“SISSI” project—aimed at clarifying several aspects of stroke occurrence in COVID-19 infection (CEUR-2021-Os-117). Patients were considered exempt from signing a written informed consent, according to the non-interventional observational nature of the study. Personal data and sensitive information were treated anonymously and according to all the principles of the Helsinki Declaration.

## Results

**Figure 2:**
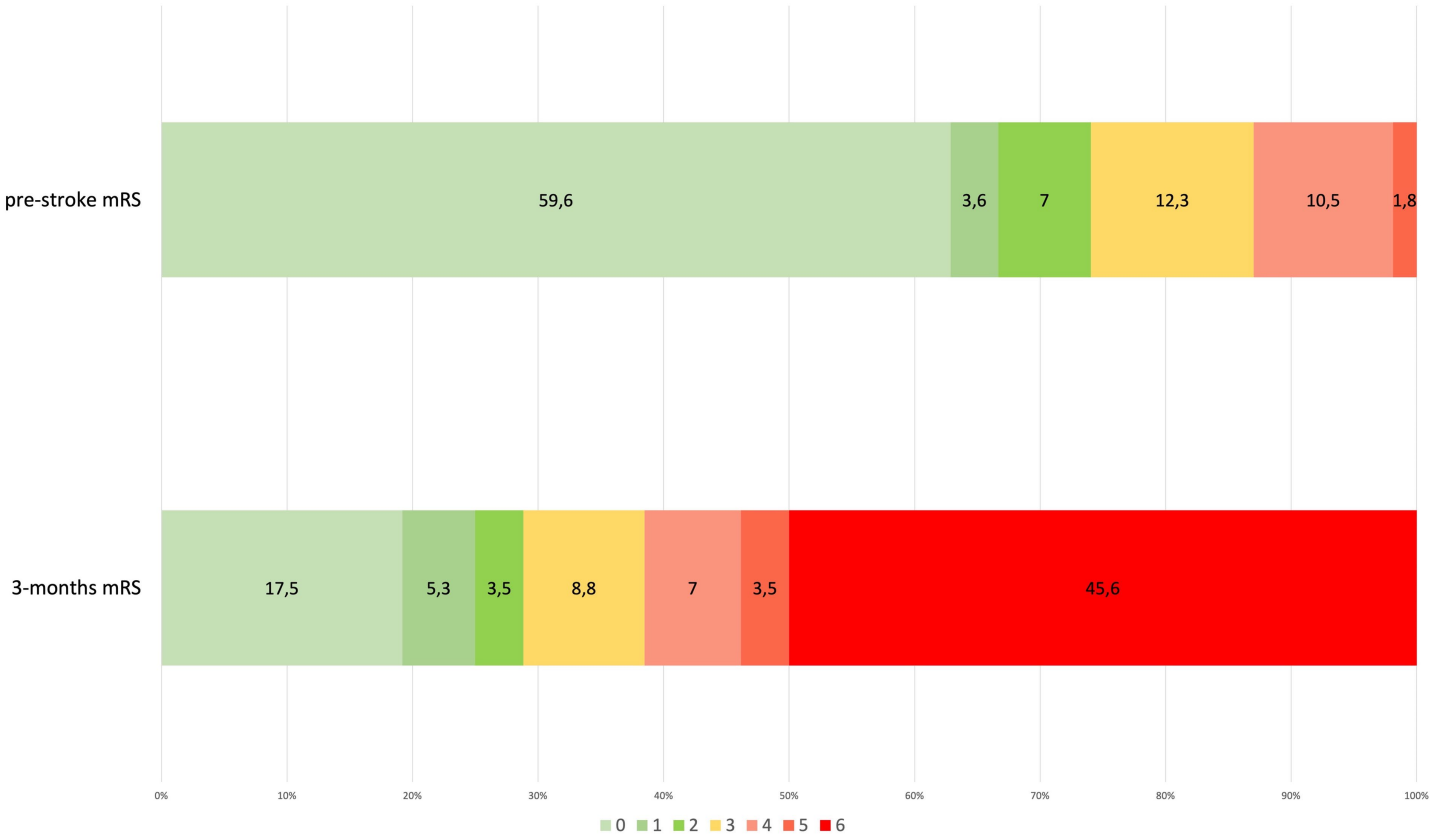
Pre-and post-stroke disability according to modified Rankin Scale (mRS) in stroke patients (percentage out of total stroke patients). Connection lines between bar histograms separate, from the left, mild or no disability (mRS 0–2), moderate disability (mRS 3), severe disability (mRS 4–5), and death (mRS 6). mRS, modified Rankin Scale.

We identified 4,105 patients hospitalized with a COVID-19 infection during the 6 months study period. Of these, 62 had a confirmed diagnosis of acute stroke. The crude rate of occurrence of stroke resulted in 1.51%. Almost all stroke patients were from Western Europe (96.8%), whereas one patient was Eastern European and another was Black. Supplementary Figure 1 shows the proportion of stroke subtypes. Notably, no subarachnoid hemorrhage (SAH) was diagnosed, and ischemic stroke subtypes: FEIS, RIS, and TIA accounted altogether for 57/62 (92%) of stroke cases (74% FEIS, 10% RIS, and 8% TIA). Figure 2 shows the outcome at 3 months of stroke patients.

Among all the hospitalized COVID-19 patients, 270 patients fulfilled the selection criteria to be part of the control sample without cerebrovascular complications. The COVID-19 with ischemic stroke group consisted of 57 patients. In Tables 1, 2 we show the clinical features of the two groups of COVID-19 patients, with and without stroke, and the statistical comparison for all the available common variables. COVID-19 patients with stroke were older, and rheumatologic or neurologic chronic disorders were more often reported in their clinical history. Stroke patients had more hypertension, ischemic heart disease, AF, congestive heart failure, and a higher proportion of anticoagulation treatment at stroke onset. Noteworthy BMI was lower in stroke patients than in the COVID-19 without stroke group. Stroke patients showed higher D-dimer levels and lower Fibrinogen levels, but no statistically significant differences in pro-inflammatory biomarkers IL6 and proADM.

**Table 1:** Common clinical baseline features of COVID-19-associated ischemic strokes and COVID-19 patients without cerebrovascular complications.

Variable (missing cases)		Overall (*N* = 327)	COVID-19 w/Stroke (*N* = 57)	COVID-19 w/o Stroke (*N* = 270)	*p*
			Mean (±SD), *N* (%), or Median [range]	Mean (±SD), *N* (%), or Median [range]	Mean (±SD), *N* (%), or Median [range]
Age (0)		68.46 (±13.65)	74.19 (±10.63)	67.25 (±13.92)	0.018
Female Sex (0)		102 (31.19)	20 (35.09)	82 (30.37)	0.588
History of neurological disorders (3)		37 (11.31)	15 (26.79)	22 (8.21)	0.037
History of rheumatologic disorders (2)		19 (5.81)	7 (12.5)	12 (4.46)	0.043
COVID-19 severity (2)		1.81 ± 0.86	1.93 ± 1.05	1.79 ± 0.81	0.3474
Moderate pneumonia to critically ill—severe COVID-19 (2)		207 (63.3)	41 (74.55)	166 (61.48)	0.092
ARDS—very severe COVID-19 (2)		75 (22.94)	19 (34.55)	56 (20.74)	0.041
COVID onset—days before stroke or admission (112)		6 [2–9]	8 [1–14.75]	6 [2–8]	0.045
Baseline antithrombotic meds (5)	SAPT	63 (19.27%)	15 (28.85%)	48 (17.78%)	0.099
	DAPT	5 (1.53%)	1 (1.92%)	4 (1.48%)	1
	Anticoagulation	44 (13.46%)	16 (30.77%)	28 (10.37%)	0.036
	Anticoagulant heparin	2 (0.61%)	0 (0%)	2 (0.74%)	1
AF (1)		48 (14.68)	20 (35.71)	28 (10.37)	<0.001
Hypertension (3)		192 (58.72)	43 (76.79)	149 (55.6)	0.005
Diabetes (1)		66 (20.18)	17 (30.36)	49 (18.15)	0.059
Smoke habit		not available	Current smoker 7 (11.3) Previous smoker 10 (16.1)	not available	-
Alcohol overuse		not available	5 (8.1)	not available	-
Hypercholesterolemia (1)		103 (31.5)	22 (39.29)	81 (30.0)	0.229
Congestive Heart Failure (2)		55 (16.82)	18 (32.73)	37 (13.7)	0.001
BMI (16)		27.88 (±5.18)	25.93 (±4.53)	28.21 (±5.22)	0.003
Ischemic heart disease (1)		23 (7.03)	10 (17.86)	13 (4.81)	0.001
Previous vascular events (4)		116 (35.47)	22 (40.0)	94 (35.07)	0.590
PLT count (81)		252,955.28 ± 111,095.67	258,981.48 ± 131,263.6	251,260.42 ± 105,058.97	0.692
INR (129)		1.24 (±0.53)	1.32 (± 0.71)	1.21 (±0.45)	0.317
aPTT (232)		1.2 ± 0.52	1.12 ± 0.28	1.26 ± 0.64	0.130
DDimer (153)		759.5 [470.75–1768.5]	2,348 [1,096–8,616.5]	647 [461–1,254]	<0.001
Fibrinogen (268)		503.19 ± 211.6	406 ± 221.87	549.35 ± 192.56	0.022
proADM (166)		1.12 ± 0.53	1.13 ± 0.48	1.12 ± 0.55	0.913
IL-6 (195)		29 [16–63.43]	52 [15–83.6]	27.3 [16–54]	0.260

Valid % are reported if missing values < 15%; SAPT, single antiplatelet therapy; DAPT, dual antiplatelet therapy; AF, atrial fibrillation; BMI, body mass index; PLT, platelet; INR, international normalized ratio; aPTT, activated prothrombin time; DD, DiDimer; Fy, fibrinogen; proADM, proadrenomedullin; IL6, Interleukin 6.

**Table 2:** Clinical baseline specific features of COVID-19 associated ischemic strokes.

Variable (missing cases)	Mean (±SD), *N* (%), or Median [range]
Ischemic stroke subtype (0)	FEIS 46 (80.7%)
	RIS 6 (10.5%)
	TIA 5 (8.8%)
Baseline NIHSS (8)	8.9 (8.4)
Concurrent vascular events (3)	None—79.6%
	PE—13.0%
	DVT/SVT—1.9%
	PE + MI—1.9%
	Other—1.9%
Reperfusion therapies (0)	None—87.7%
	IVT—12.3%
	IVT + MT—1.7%
Baseline vascular meds (0)	None—13 (23,2%)
	Antihypertensive—28 (45,2%)
	Antidiabetic—19 (30,6%)
	Lipid lowering—17 (27,4%)
	>2 drugs—26 (41,9%)
Omolateral carotid stenosis (0)	No—26.3%
	Mild—8.8%
	Moderate—10.5%
	Severe—5.3%
	Critical/occlusion—14.0%
Contralateral carotid stenosis (0)	No—26.3%
	Mild—15.8%
	Moderate—15.8%
	Severe—3.5%
	Critical/occlusion—3.5%
Leukoaraiosis—van Sweiten score (0)	0 = 19.6%
	1 = 23.2%
	2 = 19.6%
	3 = 14.3%
	4 = 23.2%
TOAST (0)	CE 10/57 (17,5)
	LAD 13/57 (22,8)
	ODE 1/57 (1,8)
	SVD 4/57 (7,0)
	UND-b 10/57 (17,5)
	UND-c 19/57 (33,3)
Managing ward at stroke onset (0)	Neurocovid 59.7%
	Medicine 29.0%
	I.C.U. 21.0%
	Infectious D. Unit 16.1%
	In-hospital stroke 35.6%

Valid % are reported if missing values < 15%; FEIS, first ever ischemic stroke; RIS, recurrent ischemic stroke; TIA, transient ischemic attack; PE, pulmonary embolism; DVT, deep venous thrombosis; SVT, superficial venous thrombosis; MI, myocardial infarction; IVT, intravenous thrombolysis; MT, mechanical thrombectomy/thromboaspiration; CE, cardioembolism; LAD, large artery disease; ODE, other determined etiology; SVD, small vessel disease; and UND, undetermined etiology.

**Figure 3:**
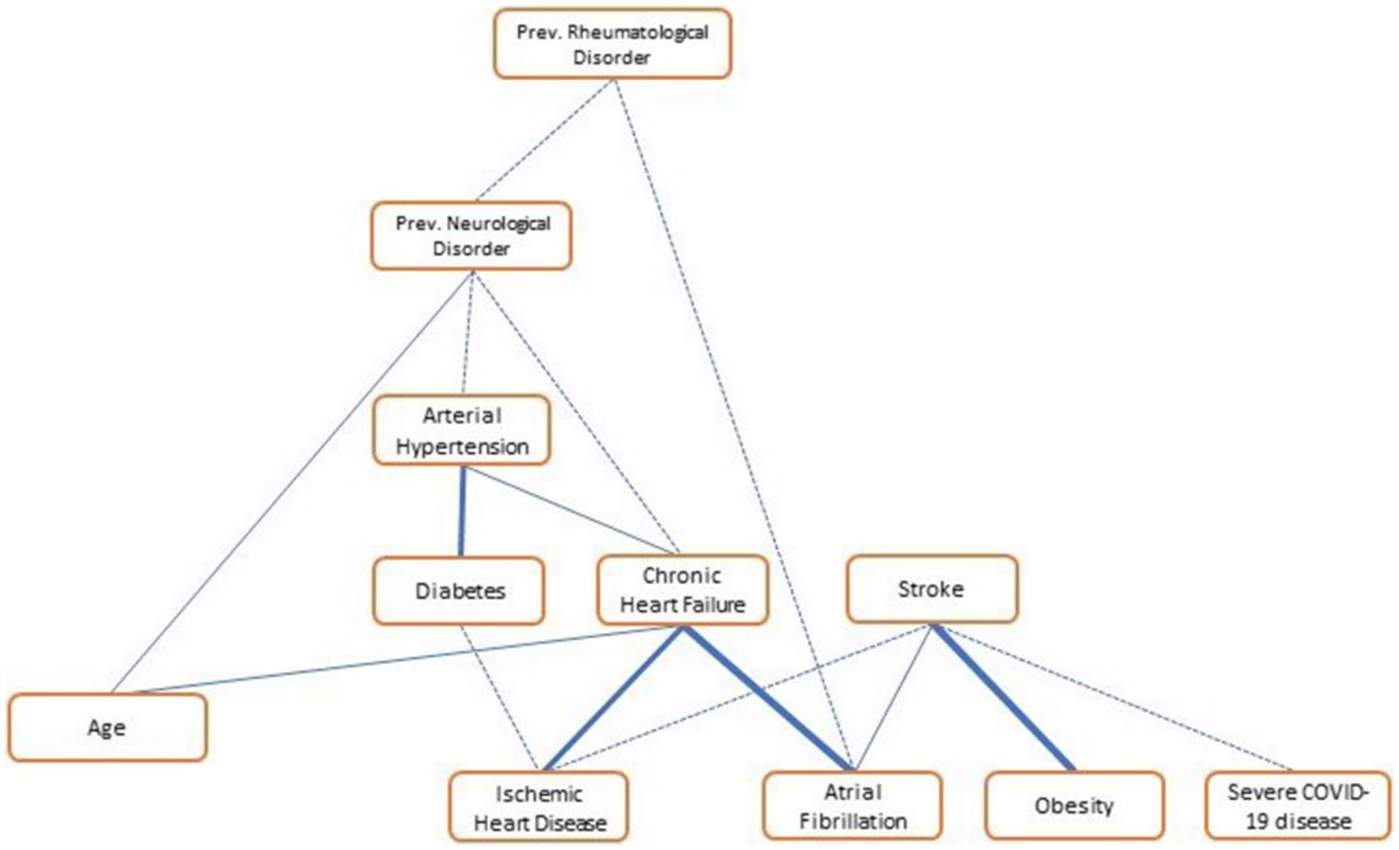
Vascular risk factor network in COVID-19 patients (*N* = 309). The network shows the link among variables of “vascular interest”; the thicker the line, the stronger the association. “Obesity,” BMI > 30; “severe COVID-19,” both ARDS and moderate to severe pneumonia.

Univariate and multivariate logistic analysis of the two groups is reported in Table 3. Atrial fibrillation and ischemic heart disease history turned out to be independent risk factors for ischemic stroke, as well as the most severe forms of COVID-19 infection. However, BMI was demonstrated to be a protective factor in COVID-19 patients. A bootstrapped network in our population of COVID-19 patients is shown in Figure 3. It depicts the mutual relationship between each vascular risk factor, with a thicker line indicating a stronger association. The direction of the risk was indicated by multivariate analysis: atrial fibrillation, ischemic heart disease, and severity of COVID-19 infection were confirmed as risk factors for ischemic stroke, while obesity was a strong protective factor.

**Table 3:** Univariate and multivariate logistic analysis of COVID-19 patients with ischemic stroke vs. COVID-19 patients without stroke.

	Univariate		Multivariate logistic	
	OR [± 95% CI]	*p*	OR [± 95% CI]	*p*
Age	1.04 [1.02–1.07]	0.001*	-	-
Sex (female)	1.24 [0.67–2.24]	0.485	-	-
Previous rheumatological dis.	3.06 [1.09–8.01]	0.025*	-	-
Previous neurological dis.	4.09 [1.94–8.50]	< 0.001*	-	-
COVID severity	1.22 [0.87–1.73]	0.263	-	-
Severe COVID	1.83 [0.97–3.64]	0.069	-	-
ARDS (very severe COVID)	2.02 [1.06–3.75]	0.029*	2.27 [1.06–4.77]	0.032
Time to admission	1.05 [1.01–1.10]	0.008*	-	-
AF	4.80 [2.44–9.41]	0.012*	3.65 [1.63–7.98]	0.001
Hypertension	2.64 [1.39–5.32]	0.004*	-	-
diabetes	1.97 [1.01–3.72]	0.041*	-	-
hypercholesterolemia	1.51 [0.82–2.73]	0.175	-	-
CHF	3.06 [1.56–5.90]	< 0.001*	-	-
BMI	0.90 [0.83–0.97]	0.006*	0.90 [0.82–0.97]	0.012
Ischemic heart disease	4.30 [1.74–10.36]	0.001*	4.590 [1.714–12.137]	0.002
Previous vascular events	1.23 [0.67–2.23]	0.488	-	-
PLT	1.000001 [0.999998–1.000003]	0.651	-	-
INR	1.40 [0.79–2.53]	0.232	-	-
aPTT	0.42 [0.10–1.19]	0.189	-	-
DDimer	1.0002 [1.0001–1.0003]	0.001*	-	-
Fibrinogen	0.997 [0.994–0.999]	0.020*	-	-
proADM	1.04 [0.49–2.02]	0.918	-	-
IL6	1.000001 [0.997–1.002]	0.999	-	-
SAPT	1.87 [0.93–3.64]	0.068	-	-
DAPT	1.30 [0.07–9.04]	0.814	-	-
Anticoagulation	3.84 [1.87–7.75]	< 0.001*	-	-
prophylactic Heparin	0.008 [− −]	0.989	-	-

OR, odds ratio; CI, 95% confidence interval; SAPT, single antiplatelet therapy; DAPT, dual antiplatelet therapy; AF, atrial fibrillation; BMI, body mass index; PLT, platelet; INR, international normalized Ratio; aPTT, activated prothrombin time; DD, DiDimer; Fy, fibrinogen; proADM, proadrenomedullin; IL6, Interleukin 6.

**Figure 4:**
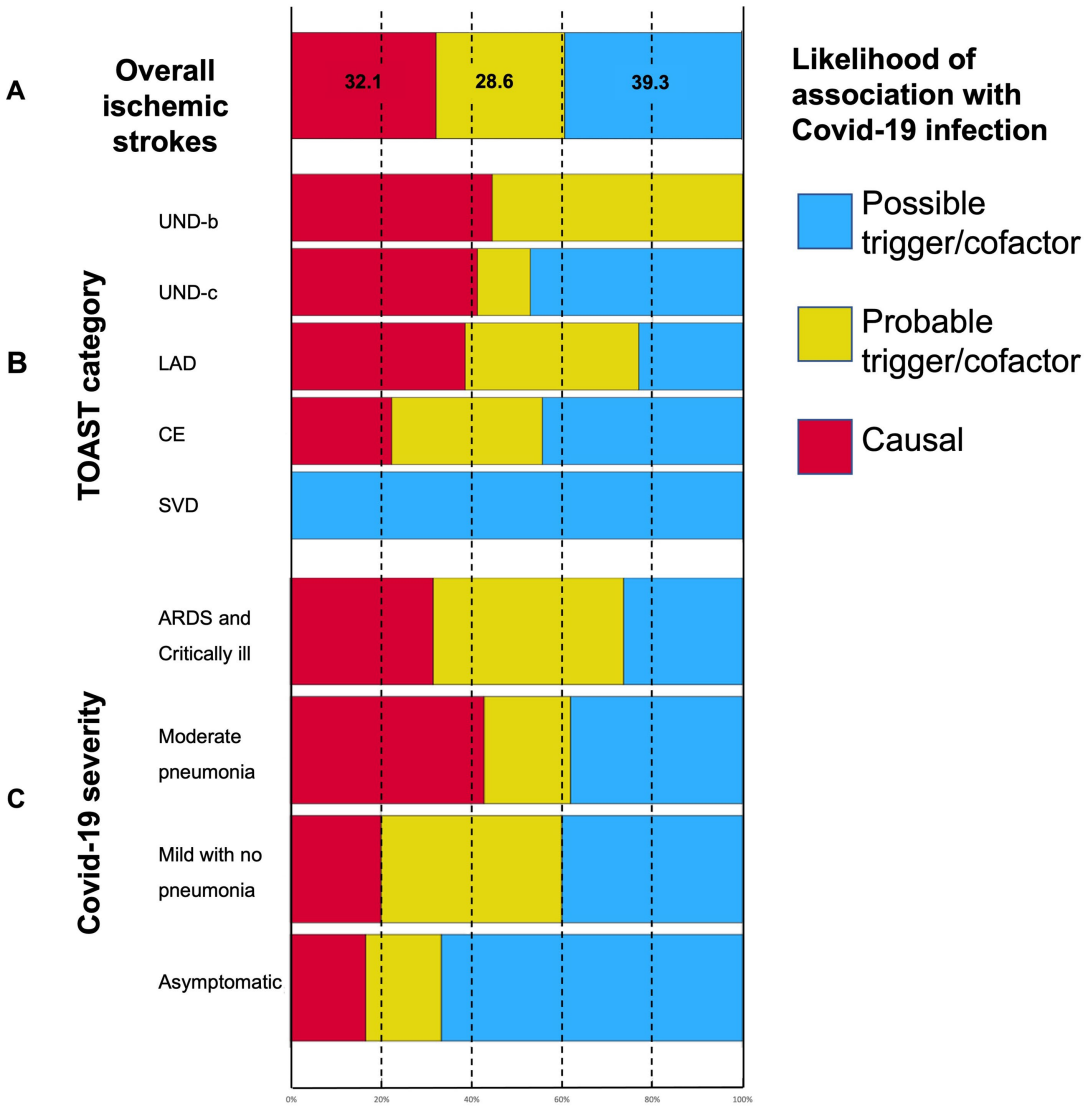
Stacked bar charts showing **(A)** the “likelihood of association” categories distribution in ischemic strokes; **(B)** the relationship between ischemic stroke mechanisms as per TOAST classification and the likelihood of stroke association with COVID-19; and **(C)** the relationship between COVID-19 severity and the likelihood of stroke association with COVID-19. See M&M for a “Likelihood of association with COVID-19” definition. UND-b, undetermined strokes because of negative evaluation; UND-c, undetermined strokes because of incomplete work-up; LAD, large artery disease; CE, cardioembolic stroke; and SVD, small vessel disease.

According to “likelihood of attribution” prespecified definitions, COVID-19 infection proved to have a causal relationship with ischemic stroke in 32.1% of events. Viral infection as a probable trigger or relevant cofactor in stroke pathogenesis was responsible for 28.6% of events, and in 39.3% of cases, it seemed to be a possible trigger only (see Figure 4A). Stacked bar charts in Figure 4 show the distribution of likelihood categories according to COVID-19 severity and TOAST classification: undetermined (UNDb and UNDc) and large artery disease (LAD) strokes, as well as severe and very severe COVID-19, have higher proportions of a causal relationship with a viral infection, although being non-statistically significant (*p* = 0.13 and *p* = 0.08 respectively).

## Discussion

This study evaluated the occurrence of acute stroke, the functional outcome, and the risk factors for acute ischemic stroke (AIS) in COVID-19 patients. We found that stroke occurred in 1.46% of hospitalized COVID-19 patients. In comparison to previous similar studies, the data suggest that, in population-based reports, stroke occurrence is consistent, and that the high variability found in other studies is likely due to selection bias in different clinical settings. Our data confirm the ischemic subtype preference and the bad outcome at 3 months as measured by mRS.

Risk analysis in our COVID-19 patients showed that atrial fibrillation, ischemic heart disease, and COVID-19 severity are independent risk factors for AIS. Infection severity has been described, and then confirmed in review articles, as a major determinant of ischemic stroke occurrence as well as of other neurological complications (38). In a recent meta-analysis, authors found a significant relative risk (RR) as high as 2.9–3.5 in severe COVID-19 cases vs. mild forms. The slight variations in RR seem to depend on the “*a priori*” definition of disease severity, but all the studies were consistent in indicating the risk factor effect direction (39). Time from COVID-19 onset to stroke occurrence varies across studies, from 0 to 19 days (40, 41). Jang et al. (42) reported that the first 3 days from COVID-19 onset were the ones with the highest incidence rate ratio (IRR) for acute stroke, 10 times higher than in non-COVID-19 patients. However, they did not provide “COVID severity” as a variable influencing COVID-19 onset to stroke occurrence. We found a significant correlation between “time from COVID-19 onset to stroke occurrence” and “COVID-19 severity/ICU setting” (*r* = 0.484, *p* < 0.001). Sluis et al. (7) found a similar correlation in their study, with AIS occurring earlier in mild/moderate COVID-19 and later in severe/critical patients. However, we are not completely sure of what was the actual infection onset, above all in less severe cases, with very mild and underreported symptoms during the incubation phase; therefore, we cannot exclude that a simple systematic error in clinical data could explain this finding.

Noteworthy, 50.8% of our AIS patients had a cryptogenic stroke. Other studies reported a higher rate of undetermined stroke etiopathogenesis (43). Although there is an undoubtful relationship between TOAST assignment and underlying causes of ischemic stroke, we argue that the role of a specific cause, such as COVID-19 infection, should be defined with criteria slightly different from TOAST. In fact, radiological and clinical features suggestive of COVID-19 related acute hyper-coagulopathy (i.e.: multifocality of brain lesions, hypointense and unstable plaques at vascular imaging indicating acute/subacute atherosclerosis, concurrent extracerebral thrombotic events, mainly PE, and occurrence despite adequate antithrombotic therapy) can fall under all the TOAST categories. Although this clarifies stroke mechanisms in several patients, it does not elucidate the causal relationship of COVID-19 infection itself. For example, a stroke due to new onset AF could either be due to preexisting cardiomyopathy or to COVID-19 induced AF; the two possibilities lead to the same attribution to cardiac embolism (CE), but in the first case COVID-19 has minor or no role, whereas in the second case, COVID-19 has at least a relevant trigger effect. Similarly, a homolateral severe carotid stenosis allows for the classification of a stroke as large artery disease (LAD), but the COVID-19 role depends more on signs of preexisting stenosis, which would indicate that COVID-19 had a minor causal/trigger role, or acute/subacute extensive atherosclerosis, which would indicate probable infection-induced active coagulopathy. Moreover, an AIS attributable to SVD is usually a minor stroke related to several known conventional vascular RFs. However, COVID-19 related endotheliopathy could theoretically cause a small deep lesion, and a causal relationship in these cases can rely more on RF profile and clinical and radiological features rather than on standard TOAST criteria. In their research paper reporting data from the Swiss Stroke Registry, Strambo et al. (4) reported a similar likelihood categorization. Our definitions differ from theirs basically in two points. First, the attribution is explicitly unbiased with TOAST classification and, second, we do not exclude “*a priori*” a triggering role of all COVID-19 infections, though this could be minor. In their experience, a causal role of COVID-19 was recognized in 24% of AIS. We found that in COVID-19 patients, 32.1% of strokes are likely to be caused by infection-induced coagulopathy, while 28.6 and 35.5% are, respectively, probably and possibly triggered by viral infection. Requena et al. (23) concluded that a minority only of strokes, 28.6% of AIS, were attributable to COVID-19 infection after a complete diagnostic workup. Altogether, these data (although few and not completely comparable) are consistent in indicating that COVID-19 infection has a causal role in nearly one-third of AIS cases, while in two-thirds, a minor triggering role is more likely. The fact that UND-b AIS (negative evaluation) shows a higher proportion of “causal” and “probable cofactor” roles than UND-c AIS (uncomplete evaluation), seems to strengthen the validity of our classification since a mix of hidden etiopathogenetic factors likely underlies the UND-c category. Finally, the analysis herein reported is a relevant frame to refine the etiopathogenesis of AIS, beyond TOAST classification, and also in other emerging subclusters of strokes, such as in AIS cases triggered by other infectious diseases and cancer-related coagulopathy.

In our population of COVID-19 patients, higher BMI values and obesity were surprisingly protective factors for AIS. Although overweight and obesity have been recognized since long ago as conventional risk factors for ischemic stroke type (44–46), in recent years evidence of an “obesity paradox” in stroke patients is increasing in the literature. In the last year, little evidence of an obesity paradox has been emerging in COVID-19 patients’ outcomes. Graziano et al. (47) demonstrated that both an increased waist circumference (WC) and bioimpedance analysis were significantly associated with the need for increased ventilator support, but not with ICU admission and—above all—not with mortality rate. In a large multicenter study, only very high BMI (> 40 kg/m^2^) was associated with higher mortality in COVID-19 cases (48). However, in that study, all the patients with metabolic syndrome had a poor outcome, independently of their BMI, raising a debate on the actual existence of an obesity paradox in COVID-19 itself. Methodological concerns around the definition of obesity have also been suggested in the general overall stroke field (49, 50). Even less data are available in the specific setting of “COVID-19 related AIS.” In a recent meta-analysis, Poly et al. (51) found that RR for mortality in COVID-19 patients increased, significantly and consistently, with classes of obesity (from 1.27 in class I to 1.92 in class III obesity), but in their secondary analysis, the “stroke subgroup” was the only one in which obesity was not significantly associated with a positive RR for mortality (1.80–0.89-3.64, *p* = 0.10). Mendes et al. (52) reported that each additional point in body mass index (BMI) significantly reduced the risk of stroke by 14%. A higher BMI has been associated in previous “general stroke” studies with a better AIS clinical outcome and lower stroke recurrence (53). However, further analysis, overcoming selection biases, did not find an association between BMI and stroke outcome, at least if case fatality at 1 month from stroke onset was considered (54). A complex interplay also exists between obesity and AF, with a U-shaped effect on RR for mortality and vascular outcome (55). Thus, the fact that AF proved an independent risk factor for AIS in COVID-19 might have contributed to the emergence of an “obesity paradox.” Tutor et al. (56) recently reviewed the literature on the obesity paradox and cardiovascular diseases and they highlighted that coronary artery disease, heart failure, and atrial fibrillation—three conditions that we found associated with ischemic stroke in COVID-19 patients—have the stronger evidence of an obesity paradox. We can hypothesize that several metabolic, endocrinological, and inflammatory changes, chronically occurring in obese patients (57), may interact in a different way, possibly protectively, in the specific setting of an AIS associated with a viral infection such as COVID-19. Noteworthy, most of these studies draw cautious conclusions, mainly because of the alternative meanings of the different measurements of overweight, other than BMI. Suk et al. (58) studied the waist-to-hip ratio and suggested that it would be a stronger risk factor for ischemic stroke, thus further complicating the interpretation of the role of BMI and obesity on vascular diseases. Finally, further studies are needed to confirm and eventually explore mechanisms of an ‘obesity paradox’ in acute stroke.

The vaccination campaigns around the world are likely to reduce the cerebrovascular risk in COVID-19 patients, mainly because of a drastic reduction in severe forms of the disease. Our data and almost all the data on stroke in COVID-19 are based on infections due to COVID-19 variants up to Delta and Omicron, and we do not completely know which is the current risk of stroke with more recent and brand-new variants. Published data on stroke reduction after vaccination are desirable. We speculate that the residual stroke risk despite vaccination might possibly be due to the triggering effect that acute infection has in patients with high vascular risk. The wider experience with influential vaccination (59) seems also to support this concept.

Our study has some strengths: the population-based setting of our study produces reliable occurrence rates and proportion of stroke subtypes, as well as a more accurate relationship (causal, concurrent factor, or apparent) between COVID-19 infection and stroke.

However, it also has some limitations. (1) We cannot exclude underestimation of stroke occurrence in COVID-19 in two specific subsets of patients: the non-hospitalized with very mild stroke symptoms (who possibly did not come to clinical attention because of stay-at-home orders) and the critical patients (whose neurological evaluation was often very hard in the COVID-dedicated-ICU setting). (2) We cannot exclude subclinical vascular events in non-critical COVID-19 patients who did not undergo specific radiological examination (e.g., head and thoracic CT, or brain MRI). (3) The selection process among COVID-19 patients without vascular complications was largely affected by the low availability of clinical data, which precluded a clear case–control matching. (4) AF is herein considered as a single variable, regardless of different subtypes: new-onset, parossistic, or chronic. (5) We had no detailed data to distinguish between Delta and Omicron effects on stroke risk, outcome, and a possible different proportion of causal vs. trigger factors of the specific virus variants themselves.

## Conclusion

Ischemic stroke is a possible thrombotic complication of nearly 1.5% of COVID-19 infections. COVID severity, atrial fibrillation, and ischemic heart disease are independent risk factors for stroke occurrence. However, obesity seems to be a protective factor, which fosters the debate about the “obesity paradox” both in COVID-19 and in cerebrovascular disease. Moreover, differently from most of the previous studies, we suggest that not all stroke events are completely attributable to COVID-19, and only one-third of them have clinical and radiological hallmarks of the so-far known “acute coagulopathy” associated with COVID-19 infection. Finally, the role of the most severe form of infection as an independent risk factor once more underlines the importance of vaccination campaigns in reducing the occurrence of vascular disease among other severe complications.

## Data Availability Statement

The raw data supporting the conclusions of this article will be made available by the authors, without undue reservation.

## Ethics Statement

The studies involving human participants were reviewed and approved by Unified Regional Ethic Committee—Friuli Venezia Giulia. Written informed consent for participation was not required for this study in accordance with the national legislation and the institutional requirements.

## Author Contributions

FJ, ES, FS, AR, CT, and MV: conceptualization. MF, DB, CC, CD, FB, GM, LN, AN, FK, SP, TS, and GB: data recovery. DB and CC: neuroimaging evaluations. ES, AR, FS, DB, CC, CD, FB, GM, LN, AN, FK, SP, TS, and GB: database systematization. AR, FS, and FJ: statistical analysis. FJ, ES, CT, and MV: article writing. CT and MV: supervision. All authors contributed to the article and approved the submitted version.

## Conflict of interest

The authors declare that the research was conducted in the absence of any commercial or financial relationships that could be construed as a potential conflict of interest.

## Publisher’s note

All claims expressed in this article are solely those of the authors and do not necessarily represent those of their affiliated organizations, or those of the publisher, the editors and the reviewers. Any product that may be evaluated in this article, or claim that may be made by its manufacturer, is not guaranteed or endorsed by the publisher.

## Supplementary material

SUPPLEMENTARY FIGURE 1 Proportion of subtypes of Covid-19 associated strokes. FEIS, first ever ischemic stroke; ris, recurrent ischemic stroke; tia, transient ischemic attack; ich, intracerebral hemorrhage.

SUPPLEMENTARY FIGURE 2 Distribution of timing (days) between Covid-19 onset and stroke occurrence.
