# Supplementary figures and images for: Ischemic strokes in COVID-19: risk factors, obesity paradox, and distinction between trigger and causal association
Source: Front Neurol. 2023 Aug 1;14:1222009. doi: 10.3389/fneur.2023.1222009 (PMC10428626; doi:10.3389/fneur.2023.1222009)

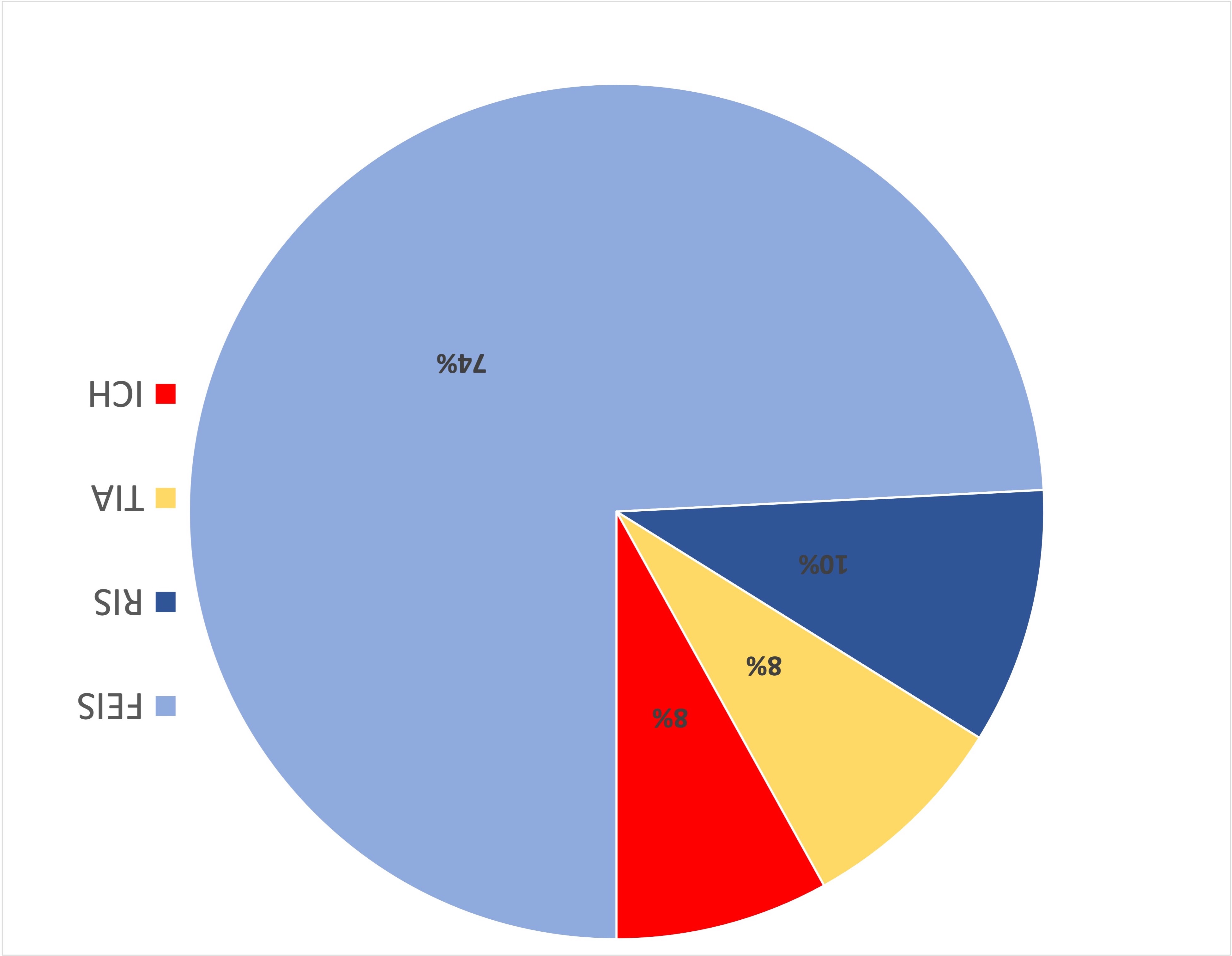

Supplement: SUPPLEMENTARY FIGURE 1 — Proportion of subtypes of Covid-19 associated strokes. FEIS, first ever ischemic stroke; ris, recurrent ischemic stroke; tia, transient ischemic attack; ich, intracerebral hemorrhage. [file Image_1.JPEG]

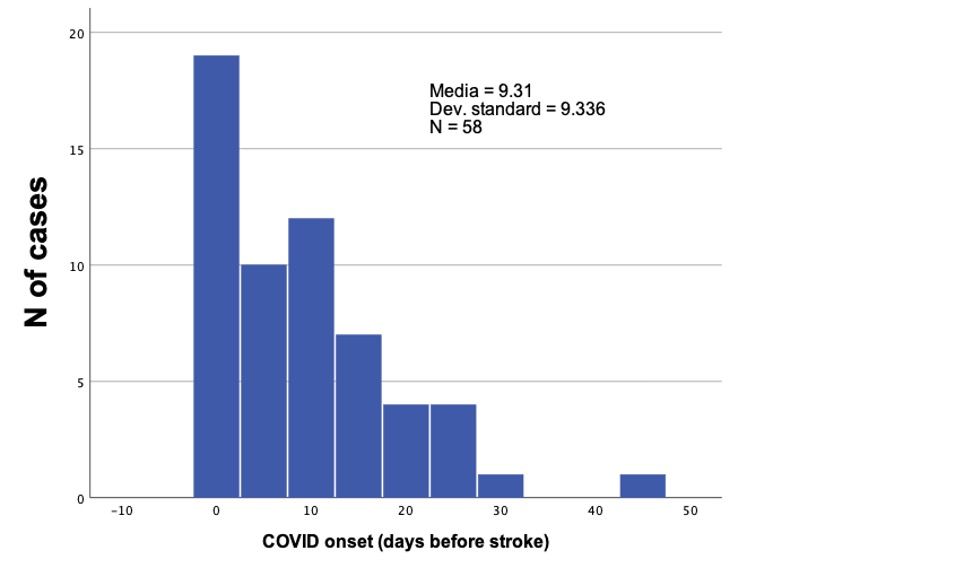

Supplement: SUPPLEMENTARY FIGURE 2 — Distribution of timing (days) between Covid-19 onset and stroke occurrence. [file Image_2.JPEG]
